# The effectiveness of Acceptance and Commitment Therapy on parental stress in parents of special children: a meta-analysis

**DOI:** 10.1186/s13034-025-00944-y

**Published:** 2025-07-21

**Authors:** Ying Guo, Haoran He, Jiajun Lan

**Affiliations:** Capital University of Physical Education and Sports, Beijing, 100191 China

**Keywords:** Acceptance and Commitment Therapy, Parents of special children, Stress, Meta-analysis

## Abstract

**Purpose:**

Through a meta-analysis, this study evaluated the effectiveness of Acceptance and Commitment Therapy (ACT) in reducing stress among parents of children with special needs. This study aimed to quantify the intervention effect and identify moderating variables, such as cultural differences and intervention parameters, to provide evidence for clinical practice.

**Methods:**

Systematic searches were conducted across the ​​PsycINFO​​, PubMed, Web of Science, and Cochrane Library databases. The inclusion criteria were as follows: (1) peer-reviewed English publications; (2) randomized controlled trials; (3) the use of ACT as the core intervention; (4) study participants being parents of children with special needs; and (5) reporting standardized effect sizes for stress symptoms. A total of 10 studies (*n* = 700) met the inclusion criteria. A random effects model was employed for the meta-analysis, and subgroup analyses were performed based on geographic distribution, intervention duration, intervention parameters, and types of children’s illnesses (neurodevelopmental disorders, chronic diseases, and critical conditions).

**Results:**

ACT significantly reduced stress symptoms among parents of special children, with a standardized mean difference (SMD =– 0.42, 95% CI– 0.72 to– 0.12, *P* = 0.0068). Subgroup analyses and meta-regression indicated a notable non-linear dose-response relationship concerning the intervention parameters. Key factors contributing to the heterogeneity of intervention outcomes included the frequency of the intervention, duration per session, overall intervention period, modalities employed, types of parental groups, and categories of children’s illnesses.

**Conclusion:**

ACT demonstrated moderate efficacy in alleviating stress among parents of exceptional children. The most significant stress reduction was observed when interventions were conducted for a minimum of 120 min per session, at least twice weekly, and over a total duration exceeding 8 weeks, particularly when ACT was integrated with other interventions for parents of children suffering from chronic and critical illnesses. Additionally, the effectiveness of the intervention in mixed-gender parent groups was significantly greater than in groups where mothers constituted over 90% of participants (*P* < 0.05). Implementing high-intensity ACT courses for populations experiencing acute stress is recommended to facilitate the rapid alleviation of stress symptoms. Concurrently, mobile health-assisted, low-density, long-term interventions are suggested for individuals dealing with chronic stress. Future research should investigate the efficacy disparities in predominantly mother-led groups and strive to develop gender-adapted ACT protocols.

**Supplementary Information:**

The online version contains supplementary material available at 10.1186/s13034-025-00944-y.

## Introduction

The stress experienced by parents of children with disabilities has increasingly been recognized as a systemic public health issue rather than merely an individual psychological phenomenon. This shift in understanding arises from the multidimensional interplay of stressors—emotional, social, and economic burdens—that undermine parental well-being and generate cascading effects on family and community systems [1]. Research indicates that healthcare professionals may often underestimate the stress levels of parents with disabled children, highlighting the need for a family-centered comprehensive assessment model [2]. Critical risk factors, such as socioeconomic difficulties, social isolation, and a lack of professional support, interact with protective factors, such as family and community support, to influence the dynamics of stress [3]. The COVID-19 pandemic further exacerbated these stresses, as challenges related to remote education and the balancing of professional and family responsibilities significantly heightened levels of anxiety and depression [4], although flexible work policies may have alleviated some of this pressure [5].

The stress experienced by parents of children across various disease spectrums exhibits heterogeneous characteristics. For example, parents of children with cerebral palsy (CP) must navigate the physical burdens associated with motor rehabilitation and long-term care [6]; families of children with autism spectrum disorder (ASD) contend with social pressures stemming from deficits in social skills and behavioral management [7]; meanwhile, parents of children with cancer and chronic illnesses find themselves ensnared in an ‘uncertainty vortex’ characterized by the need to monitor treatment side effects and fluctuating survival rates [8]. These diverse sources of stress initiate a biopsychosocial cascade: chronic elevations in cortisol can disrupt the regulation of the hypothalamic-pituitary-adrenal (HPA) axis [9], and negative cognitive biases may intensify familial conflicts [10]. Notably, parents of children with CP exhibit significantly higher rates of depression and anxiety compared to parents of typically developing children, with these outcomes influenced by the child’s functional level and the family’s socioeconomic status [11]. In families of children with cancer, parental stress and the child’s pain levels reinforce each other in a bidirectional manner, and the stress experienced by parents of adolescents with ASD may persist into the child’s adulthood [12, 13]. The specificity of this stress is evident in its chronicity (prolonged duration), multidimensionality (interconnected medical, economic, and emotional factors), and uncontrollability (unpredictable disease progression), which render conventional stress management strategies ineffective.

Although mindfulness-based stress reduction (MBSR) has been widely adopted, its decontextualized nature does not adequately address the dynamic needs of families with exceptional children, such as during emotional meltdowns in autism or pre-treatment stress in cancer [14]. Furthermore, insufficient cultural adaptation limits its effectiveness; for instance, interventions targeting African American families with autism spectrum disorder (ASD) often overlook culturally specific factors [15]. While cognitive behavioral therapy (CBT) demonstrates broad applicability, it may evoke a sense of ‘rational oppression’ in contexts involving medical ethical dilemmas or social discrimination, which can lead to high dropout rates [16]. Neuroimaging studies have revealed a potential ‘dual-network abnormality’ in parents of special children: hyperactivation of the default mode network (DMN), which may be associated with negative rumination, paired with hypoactivation of the salience network (SN), which could impair the identification of positive resources. This suggests that traditional CBT’s cognitive restructuring strategies may be inadequate for addressing their emotion- and value-driven needs [17–19].

Acceptance and Commitment Therapy (ACT) offers a multidimensional intervention pathway for parents of children with special needs by reconstructing stress-related cognitions—specifically, the responses to perceived threats against personal values—and enhancing psychological flexibility, defined as the capacity to maintain mindful awareness, open acceptance, and value-guided action [20]. Behaviorally, ACT shifts the parental role from passive coping to active meaning-making, enhancing self-efficacy and improving mental health outcomes through value-driven actions. Socially, the cognitive defusion techniques employed in ACT, such as “labeling externalization,” effectively disrupt the negative cycles of shame and self-blame, thus reconstructing self-identity [21]. Cross-cultural studies have demonstrated the cultural sensitivity of ACT effectiveness. For instance, culturally adapted ACT interventions have significantly reduced anxiety and depression in Hispanic caregivers of children with ASD [22], and modifications in the Korean RUBI program have shown advantages in behavioral management [23]. However, heterogeneity analyses reveal that ACT’s effectiveness is moderated by the visibility of stressors and levels of social support; effect sizes in ASD families are significantly lower than those in families with children who have hearing impairments [21, 24]. Although existing evidence supports a moderate to high efficacy of ACT, methodological heterogeneity (e.g., intervention durations ranging from 4 to 12 weeks and differences in individual versus group formats) and operationalization deficiencies of cultural adaptation mechanisms have limited these conclusions’ generalizability and clinical translatability. Therefore, conducting a meta-analysis of ACT interventions on parental stress holds significant academic and practical value. ​​Meta-analysis is uniquely positioned to systematically integrate evidence from heterogeneous studies, quantify the overall effect size, and identify key moderating variables (e.g., cultural differences, intervention duration). This approach provides high-level evidence crucial for evidence-based practice.

## Materials and methods

### Study design

This study constitutes a systematic review of randomized controlled trials (RCTs) conducted by the Preferred Reporting Items for Systematic Reviews and Meta-Analyses (PRISMA) guidelines [25]. Before screening the search results, the protocol was registered with the International Prospective Register of Systematic Reviews (PROSPERO; registration number: CRD420251005277) and executed in alignment with the PRISMA statement.

### Study inclusion criteria

Randomized controlled trials (RCTs) meeting the PICOS criteria were included: ​​Participants (P)​​ targeted parents (regardless of gender, ethnicity, or socioeconomic background) of children with special needs (e.g., disabilities, chronic illnesses, neurodevelopmental disorders); ​​Intervention (I)​​ involved Acceptance and Commitment Therapy (ACT) with ≥ 1 session; ​​Comparator (C)​​ included blank controls (e.g., waitlist/treatment-as-usual) or active conventional treatments (e.g., CBT); ​​Outcomes (O)​​ required standardized measurement of parental stress (primary/secondary outcome); ​​Study design (S)​​ was restricted to RCTs. Only full-text English publications in peer-reviewed journals were eligible. Systematic reviews were excluded from meta-analysis but manually screened for citations and potential eligible studies. Exclusion criteria encompassed narrative/preclinical studies, duplicates, editorials, grey literature, conference abstracts, protocols, non-RCT designs, and studies without validated stress scales.

### Search strategy

This meta-analysis aimed to assess the effect of Acceptance and Commitment Therapy on stress among parents of exceptional children and adolescents. Searches were conducted in the PubMed, Web of Science, PsycINFO, and Cochrane Library databases to identify relevant and comprehensive studies, concluding on February 18, 2025. The search terms employed included: (“Acceptance and Commitment Therapy” OR “ACT”) AND (“Stress” OR “Stress symptoms”) AND (Parents OR Mothers) AND (“children with cerebral palsy” OR “children with CP” OR “children with Autism Spectrum Disorder” OR “children with autism” OR “children with chronic conditions” OR “children with chronic illness” OR “children with acquired brain injury” OR “children with hearing impairment” OR “children with asthma” OR “children with cancer” OR “special needs children”) AND (“Controlled Trial” OR “Randomized Controlled Trial” OR “RCT” OR “Clinical Trial” OR “Controlled Study” OR “Comparative Study” OR “Placebo-Controlled Trial”). Additionally, reference lists were examined to identify further eligible studies. Detailed search strategies can be found in the supplementary file.

### Study selection process

The search results were imported into Zotero 7.0 for effective management, and duplicate records were initially removed. Two independent reviewers subsequently screened the titles and abstracts to exclude studies that did not meet the inclusion criteria. Full texts were obtained and assessed in detail for studies that potentially met the requirements. In instances of disagreement between the two reviewers regarding study inclusion, a third independent reviewer was consulted to reach a consensus, thereby ensuring objectivity and accuracy in the screening process. Data extraction was conducted independently by two reviewers, with any discrepancies resolved through the involvement of a third reviewer.

### Data synthesis

Data analysis was conducted using R software, utilizing the meta, metafor, diameter, and ggplot2 packages. For continuous outcome measures, the mean difference (MD) or the standardized mean difference (SMD) was selected as the effect size, depending on the consistency of the measurement tools across studies. The SMD was calculated using Hedges’ g, with effect sizes categorized as small (g = 0.2), medium (g = 0.5), and large (g = 0.8) [26]. The pooled effect size was computed by weighting each study’s effect size by the inverse of its variance, primarily employing the DerSimonian-Laird random effects model; a fixed effects model was considered when heterogeneity was low. Heterogeneity was assessed using the chi-square test (with significance defined as *p* < 0.10) and the I² statistic (with I² >50% indicating significant heterogeneity) [27]. Publication bias was examined by visually inspecting a funnel plot and performing the Egger regression test for asymmetry, supplemented by a sensitivity analysis using the Trim-and-Fill method [28]. Furthermore, subgroup analyses, sensitivity analyses, and influence diagnostics (using functions such as Metainf and InfluenceAnalysis) were conducted to explore the impact of individual studies on the overall effect and assess the findings’ robustness. To evaluate potential moderating effects, meta-regression analysis was performed using a random effects model based on the restricted maximum likelihood (REML) method, and bubble plots were generated to visually present the relationship between moderating variables and effect size [29].

### Risk of bias (quality) assessment

The quality of the included studies was assessed using the Cochrane Risk of Bias tool, which encompasses several domains: random sequence generation, allocation concealment, blinding of participants and personnel, blinding of outcome assessment, incomplete outcome data, selective reporting, and other potential biases [30]. Two reviewers conducted quality assessments independently, and any discrepancies were resolved through consultation with a third reviewer. To enhance the visualization and interpretability of the evidence, the overall findings were graphically displayed and analyzed using the EVDMAP platform (https://www.pymeta.com/evdmap/), thereby clearly representing the distribution and quality levels of the studies [31].

## Results

### Study selection

**Fig. 1:**
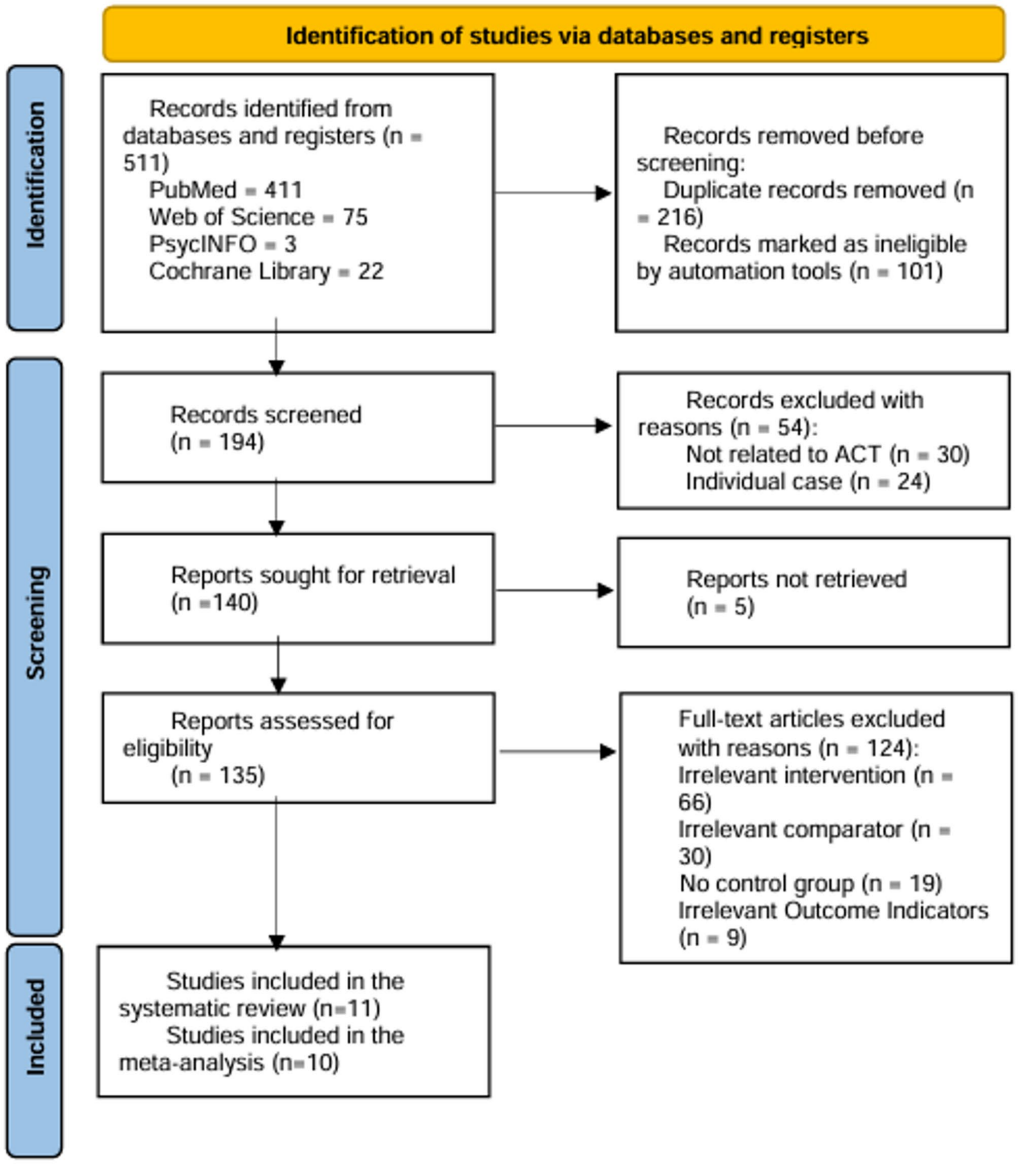
Flow diagram of the selection process

Five hundred eleven articles were identified from the four databases, with 216 duplicates. After screening the titles and abstracts of 194 articles, 54 were excluded for failing to meet the inclusion criteria. The remaining 140 articles underwent a full-text review. Detailed reasons for the exclusion of 124 articles are presented in Fig. 1. Ultimately, 11 studies were included in this systematic review [32–42].

One of the 11 studies in the systematic review was excluded from the meta-analysis due to heterogeneity and bias assessment.

### Risk of bias of included studies

**Fig. 2:**
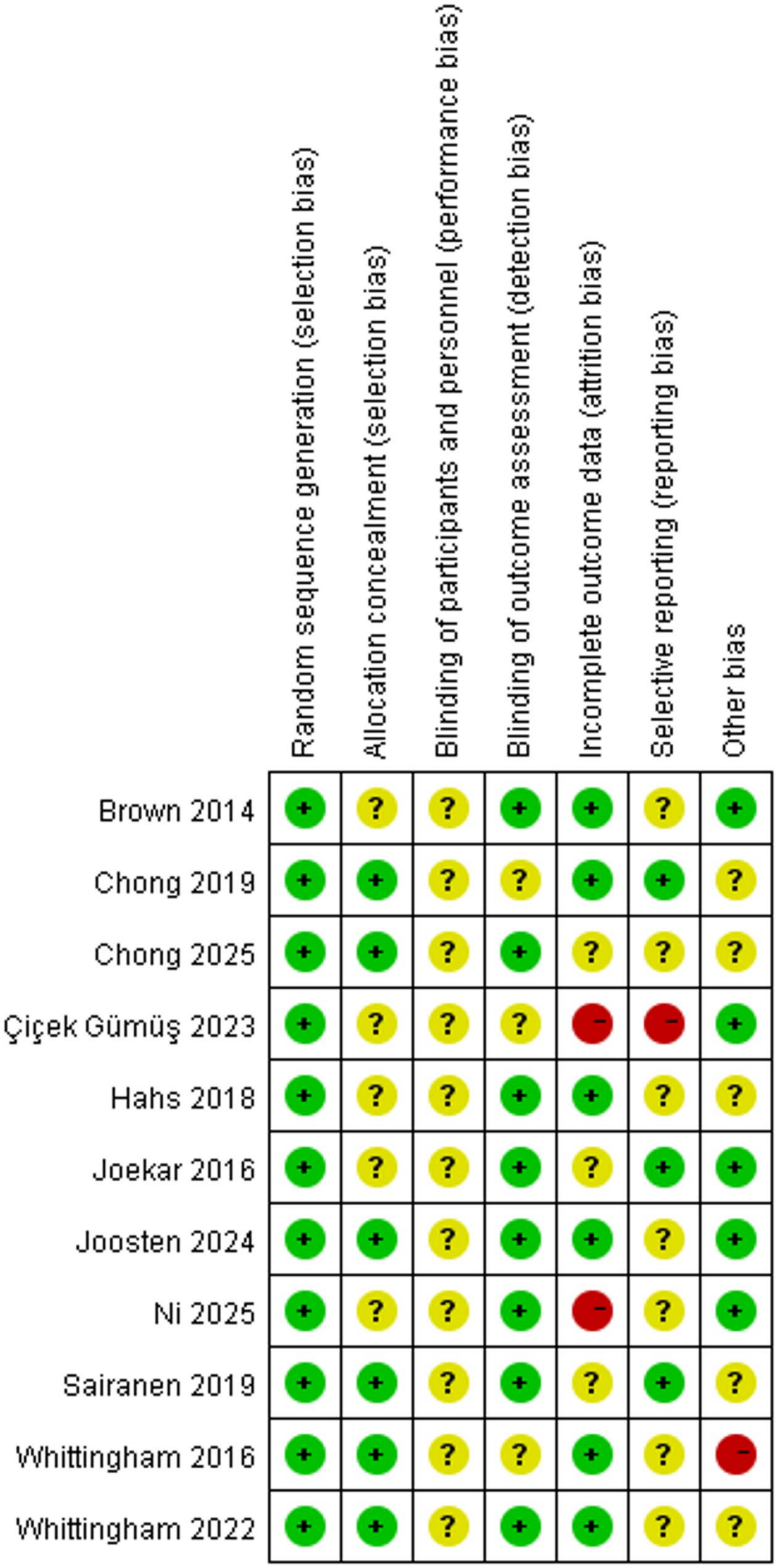
Risk of bias summary

**Fig. 3:**
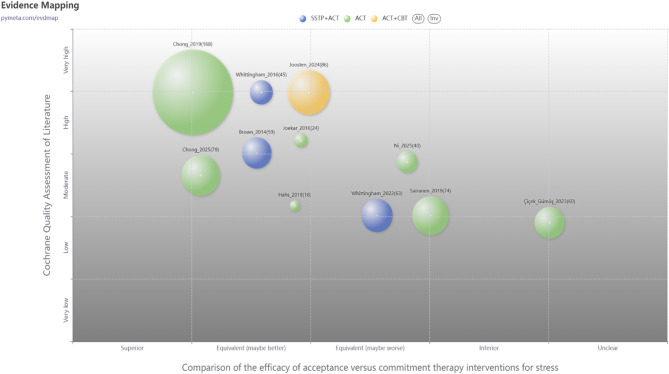
Evidence mapping of Acceptance and Commitment Therapy interventions for stress

All included studies adequately described the generation of random allocation sequences, indicating a low risk of selection bias associated with sequence generation. Six trials provided specific details regarding sample allocation concealment and were thus rated low risk [32, 35–38, 40]. In contrast, the remaining studies lacked comprehensive descriptions and transparent information regarding these procedures, resulting in a classification of unclear risk. Regarding performance bias, the nature of the studies and the number of personnel involved posed challenges for coaches or researchers in maintaining blinding. Nonetheless, in studies evaluating the effects of ACT on stress among parents of children and adolescents with special needs, blinding of outcome assessment was deemed crucial for trial integrity. Attrition and reporting bias were significantly influenced by three studies [40–42]. Figures 2 and 3 provide a summary and an evidence map of the risk of bias, respectively.

### Study characteristics

The included studies primarily investigated health interventions aimed at alleviating parental psychological stress, specifically focusing on the application of ACT across diverse populations. The subjects predominantly consisted of parents of children with special needs, including those with cerebral palsy (CP), autism, asthma, and cancer, among others. The intervention designs often integrated ACT with additional therapeutic approaches, such as Stepping Stones and Cognitive Behavioral Therapy, and compared these combined interventions with control groups. The duration and frequency of the interventions varied across studies, typically ranging from one to two sessions per week, with session lengths of 35 to 120 min and intervention periods lasting between four to ten weeks. Regarding assessment tools, most studies employed the Depression Anxiety Stress Scales for evaluating psychological health. Sample sizes ranged from 9 to 84 participants, with studies conducted in countries including Australia, China, the United States, Turkey, and Sweden, involving participants of various ages and cultural backgrounds (see Table 1).

**Table 1:** Characteristics of the studies in the systematic review and meta-analysis

Author/Year	Author’s country	Sample size (T/C)	Age range	Subject type	Intervention design (T/C)	Exercise prescription	Evaluation tools/content
Whittingham 2022 [32]	Australia	25/29	30–45	parents of children with a diagnosis of CP	Stepping Stones with Acceptance and Commitment Therapy / the wait-list control group	120 min/session, 1 time/week, 10 weeks	DASS
Brown 2014 [33]	Australia	30/29	39.14 ± 1.88	parents with pediatric-acquired brain injury	Acceptance and Commitment Therapy + Standardized Stepping Stones Triple P / Care As Usual	90 min/session, 1 time/week, 10 weeks	DASS
Joekar 2016 [34]	Iran	12/12	34.96 ± 4.26	Mothers of children with high-functioning autism	Acceptance and Commitment Therapy/treatment as usual	90 min/session, 1 time/week, 8 weeks	DASS
Sairanen 2019 [35]	Sweden	37/37	34.8–51.2	parents whose children have chronic conditions	web-based Acceptance and Commitment Therapy / Control Group	35 min/session, 1 time/week, 10 weeks	DASS
Chong 2019 [36]	China	84/84	38.40 ± 5.90	parents of children with asthma	Acceptance and Commitment Therapy / Control group	90 min/session, 2 times/week, 4 weeks	DASS
Joosten 2024 [37]	Netherlands	42/44	41.9 ± 7.5	parents of children with cancer	Cognitive Behavioral Therapy and Acceptance and Commitment Therapy / Control group	120 min/session, 1 time/week, 7 weeks	PROMIS and DT-P
Whittingham 2016 [38]	Australia	23/22	38.73 ± 7.21	parents of children with a diagnosis of CP	Stepping Stones Triple P and Acceptance and Commitment Therapy / the wait-list control group	120 min/session, 2 times/week, 6 weeks	DASS
Whittingham 2016 [38]	Australia	23/22	38.73 ± 7.21	parents of children with a diagnosis of CP	Stepping Stones Triple P and Acceptance and Commitment Therapy / Stepping Stones Triple P	120 min/session, 2 times/week, 6 weeks	DASS
Hahs 2018 [39]	America	9/9	45.5 ± 6.14	parents of children with autism spectrum disorder	Acceptance and Commitment Therapy / Control Group	120 min/session, 2 times/week, 1 week	ISS
Chong 2025 [40]	China	39/39	41.3 ± 11.0	parents of children with eczema	Acceptance and Commitment Therapy + eczema management education/treatment as usual	120 min/session, 4 times/week, 4 weeks	DASS
Çiçek Gümüş 2023 [41]	Turkey	30/30	36–51+	parents with special needs children	Acceptance and Commitment Therapy / routine training sessions	60 min/session, 1 time/week, 6 weeks	DASS
Ni 2025 [42]	China	20/20	35.78 ± 4.84	parents of children with autism spectrum disorder	Acceptance and Commitment Therapy/treatment as usual	120 min/session, 1 time/week, 8 weeks	PSI-SF

*RCT* Randomized Controlled Trial,* T* Intervention Group,* C* Control Group,* ACT* Acceptance and Commitment Therapy,* CBT* Cognitive Behavioral Therapy,* DASS* Depression Anxiety Stress Scales,* PROMIS* Patient-Reported Outcomes Measurement Information System,* DT-P* Distress Thermometer for Parents,* ISS* Inflexible Psychological Stress Scale,* PSI-SF* Parenting Stress Index-Short Form;

### Meta-analysis

**Fig. 4:**
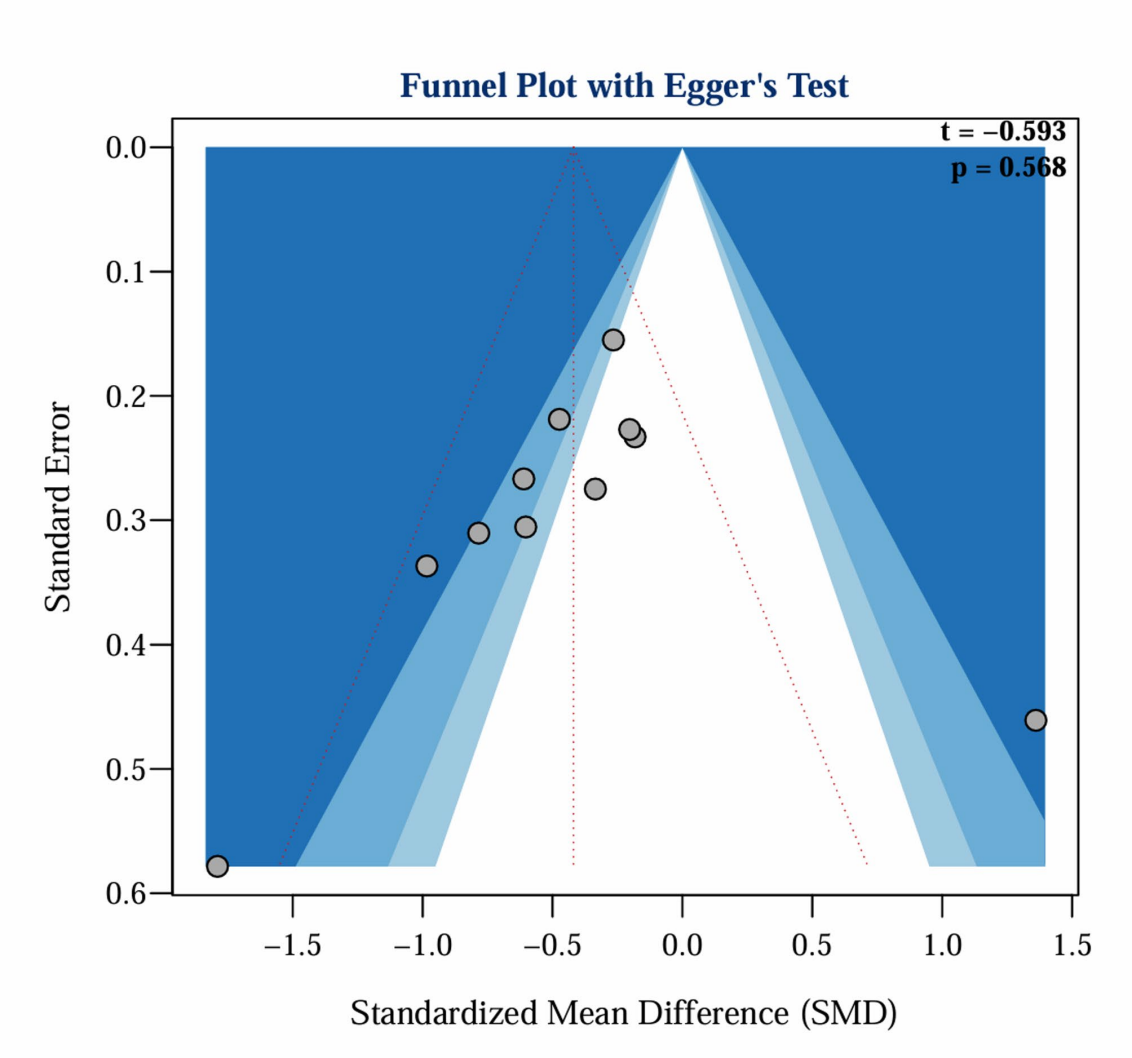
Funnel plot and Egger test for publication bias

This study systematically evaluated the effect of ACT on stress symptoms among parents of children with special needs by including 11 studies [32–42]that assessed stress in 760 parents. The SMD was selected as the appropriate effect size since different standardized scales were used across studies to measure parental stress (e.g., DASS-21, ISS, PSI-SF).​​The heterogeneity among studies was high (I² = 89.7%, *P* < 0.001), prompting analysis using a random effects model. The results indicated that ACT constituted a statistically significant but heterogeneous intervention for reducing stress among parents of children with special needs (SMD =– 0.78, 95% CI =– 1.29 to– 0.28, *P* = 0.002). Given the high heterogeneity (I² >50%, I² = 89.7%, *P* < 0.000), the Egger regression test was performed to evaluate the presence of publication bias. The Egger regression, a statistical method designed to detect publication bias in meta-analyses, was primarily employed to assess whether the results were influenced by minor sample effects [43]. The Egger’s regression test showed no statistically significant publication bias (t =– 1.87, *P* = 0.091 > 0.05). However, the observed extreme effect size in Çiçek Gümüş et al.‘s study (SMD =– 5.74) warrants caution when interpreting these findings, as such outliers may indicate methodological heterogeneity or sampling anomalies [41], necessitating a sensitivity analysis to investigate the source of the publication bias. Sensitivity analysis, a statistical method employed in meta-analyses to evaluate the robustness and reliability of results by determining the impact of modifying key parameters or excluding specific studies [44], revealed that the study by Çiçek Gümüş et al. significantly affected the publication bias [41](see Supplementary Figures). Notably, sensitivity analysis showed that the overall SMD decreased from − 0.78 to − 0.42 after excluding the study by Çiçek Gümüş et al. Following the exclusion of this study, the Egger test no longer indicated any bias (t = − 0.593, *P* = 0.568), as illustrated in Fig. 4. A review of the original article by Çiçek Gümüş et al. indicated that by using the DASS-21 to assess stress symptoms, the experimental group’s scores decreased from 17.50 ± 3.44 to 4.70 ± 1.70. In contrast, the control group’s post-intervention score remained at 17.50 ± 2.61. Given that effect sizes in psychological interventions typically range from 0.2 to 0.8, this value is considerably outside the acceptable range, indicating potential anomalies in the data [41].

**Fig. 5:**
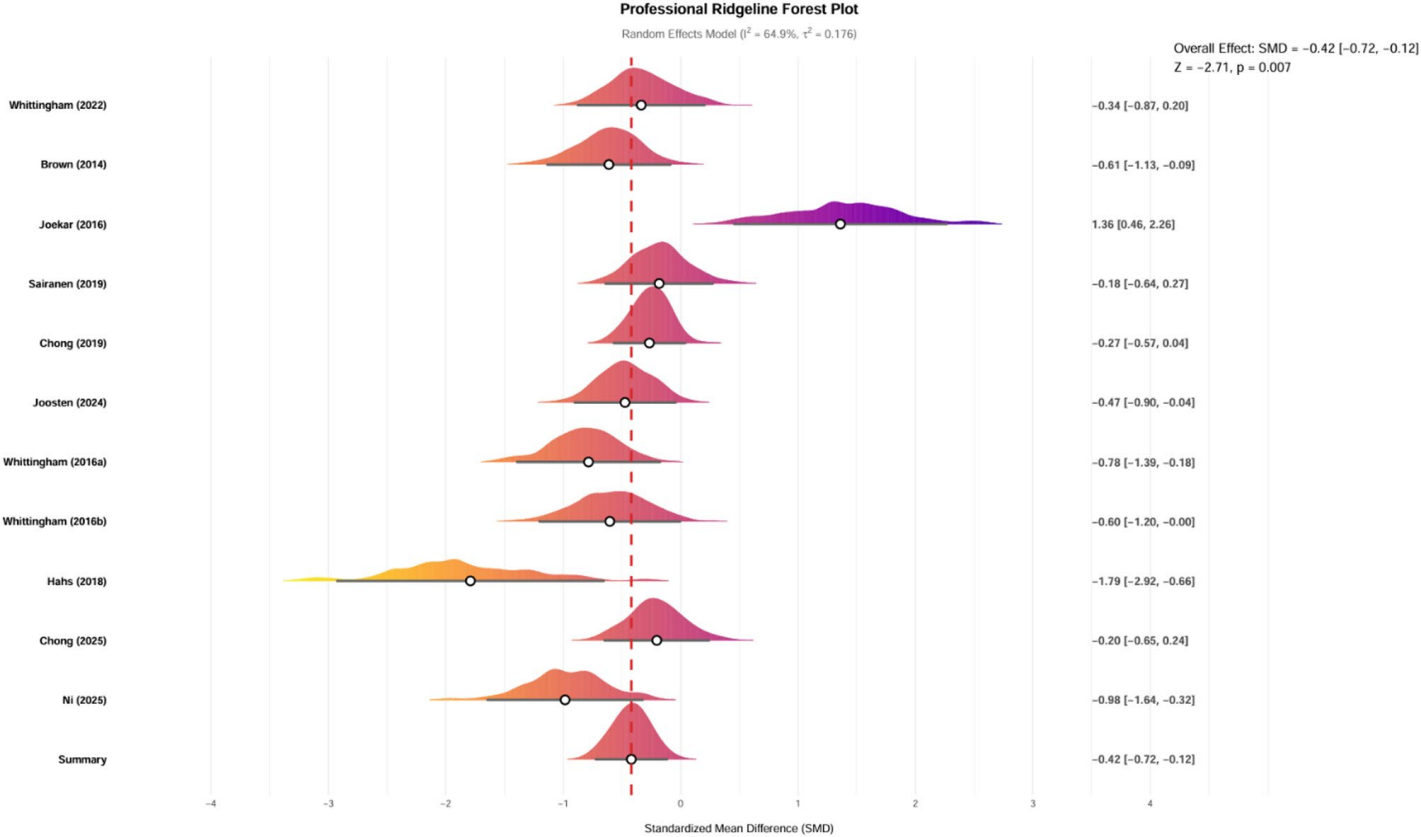
Forest plot of the effect of Acceptance and Commitment Therapy on stress among parents of exceptional children

The final analysis encompassed ten studies evaluating the impact of ACT on depression in a sample of 700 parents. The heterogeneity among the studies was moderate, with an I² value of 64.9% (*P* = 0.0015), prompting the application of a random-effects model. The results indicated that ACT significantly alleviated depression among parents of children and adolescents with special needs (SMD =– 0.42, 95% CI– 0.72 to– 0.12， *P* = 0.0068), as illustrated in Fig. 5.

### Subgroup analysis

**Fig. 6:**
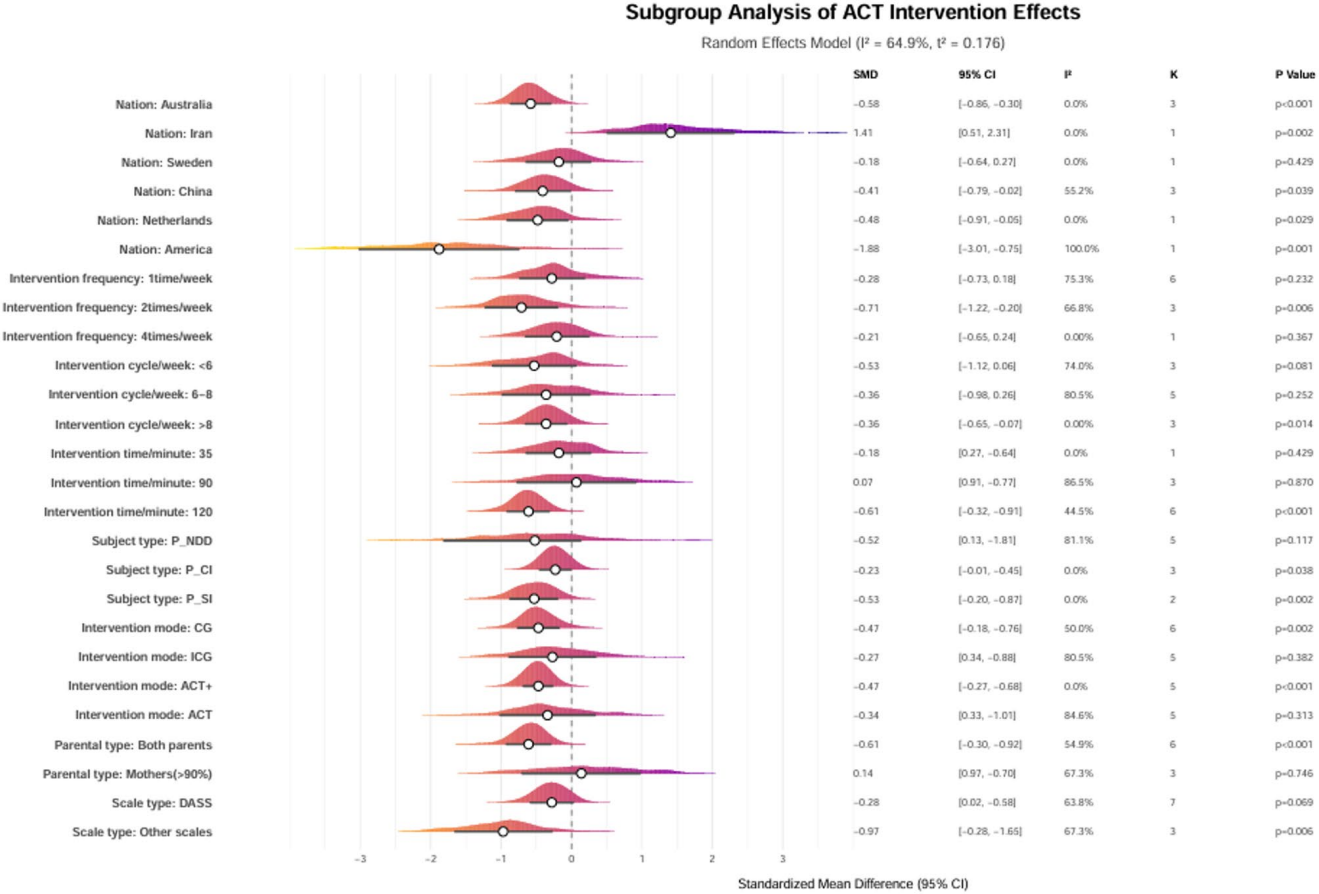
Forest plot of subgroup analysis for the effect of Acceptance and Commitment Therapy on parental stress among parents of exceptional children and adolescents

To further explore the factors influencing the efficacy of ACT on stress among parents of children with special needs, subgroup analyses were conducted based on various factors, including the types of children’s conditions, parental roles, intervention modalities, country/region, duration of intervention, frequency of intervention, and overall intervention period. No subgroup analysis was performed based on age since most parents were between 30 and 40 years old. The subgroup analysis revealed significant heterogeneity in the effects of ACT on parental stress. Regarding intervention parameters, the optimal effect was observed with a regimen of two weekly sessions for more than eight weeks, lasting 120 min (SMD ranging from − 0.71 to -0.61, *p* ≤ 0.006). Furthermore, ACT combined with other intervention measures (SMD = -0.47, *p* < 0.001) proved more effective than ACT alone. Among the participants, parents of children with severe illnesses (SMD = -0.53, *p* = 0.002) and those with chronic conditions (SMD = -0.23, *p* = 0.038) exhibited significant benefits. In contrast, interventions involving both parents (SMD = -0.61, *p* < 0.001) were more advantageous than those involving only mothers. Notable differences were observed in the sensitivity of measurement tools, with non-DASS scales detecting a more prominent effect (SMD = -0.97 vs. -0.28). The heterogeneity among studies was high (I² ranged from 44.5 to 100%), particularly within the neurodevelopmental disorder subgroup (I² = 81.1%) and the moderate intervention duration subgroup (6–8 weeks, I² = 80.5%). This indicates that future studies should standardize intervention protocol designs and enhance research on cross-cultural adaptation, as illustrated in Fig. 6.

### Meta-regression

The meta-regression analysis of ten randomized controlled trials, conducted using a random effects model, revealed that ACT moderately reduces stress among parents of children and adolescents with special needs (SMD = -0.42). The analysis of moderating effects indicated that race (β = 1.13, *p* = 0.061) and caregiver type (β = 0.70, *p* = 0.062) exhibited marginal significance, suggesting that parents and dual-parent participation may yield better outcomes. However, the parameters of intervention dosage (frequency, duration, and overall period) and the type of children’s diseases did not demonstrate significant moderating effects (*p* > 0.15). Notably, the race variable completely explained between-study heterogeneity. This may indicate geographical sampling bias, given that a disproportionate number of studies were conducted in high-income countries. This underscores the critical importance of cultural adaptation on intervention outcomes. Although ACT demonstrated cross-diagnostic stability, the heterogeneity analysis revealed existing methodological limitations, including inadequate standardization of dosage reporting, a lack of non-Western samples, and missing mechanistic variables.

**Fig. 7:**
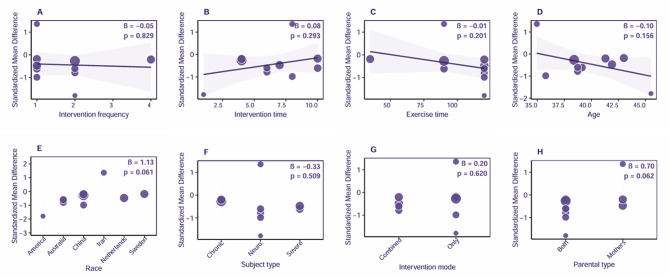
Bubble plot of the meta-regression analysis for the effect of Acceptance and Commitment Therapy on stress among parents of exceptional children and adolescents

The subgroup analysis and meta-regression results indicated that ACT moderately reduced stress among parents of children with special needs (SMD = -0.42), albeit with significant heterogeneity. Cultural characteristics of the regions emerged as a key moderating factor, with substantial effects observed in high-income countries such as Australia, China, and the USA, while an anomalous impact was noted in Iran. Furthermore, intervention protocols that involved dual-parent participation, combined therapy approaches, and longer, high-frequency interventions (two sessions per week for more than eight weeks, 120 min per session) were the most effective. The stability of the effect was evident across diagnostic categories, with both severe and chronic disease groups benefiting; however, there was a notable difference in the sensitivity of measurement tools, as non-DASS scales demonstrated effect sizes three times higher. The current evidence is limited by methodological heterogeneity, a predominance of Western samples, and deficiencies in cultural adaptation (see Fig. 7).

## Discussion

This meta-analysis confirmed that ACT moderately reduces stress among parents of children with special needs (SMD =– 0.42, 95% CI– 0.72 to– 0.12, *P* = 0.0068). One study focusing on parents of children with autism spectrum disorder found that an ACT-based parenting program not only significantly reduced parenting stress but also improved various psychological health indicators. Furthermore, the intervention enhanced parental confidence and reduced children’s emotional and behavioral problems [42]. During the COVID-19 pandemic, both group-based online and face-to-face ACT interventions demonstrated improvements in parental stress and mental health, with effect sizes ranging from small to moderate. This finding suggests that ACT exhibits strong adaptability and feasibility across different delivery formats [45]. Additionally, for adults experiencing moderate levels of stress, even self-help formats without therapist support enabled significant stress reduction, indicating that self-help approaches may serve as an economical and convenient stress management strategy [46].

Subgroup analyses and meta-regression revealed a significant nonlinear dose-response relationship concerning the intervention parameters. The optimal stress alleviation effect of ACT combined with other interventions was observed when each session lasted at least 120 min, was conducted at a frequency of at least twice per week, and extended over a period exceeding 8 weeks, particularly among parents of children with chronic or severe illnesses. Notably, the effect size in mixed-gender parent groups was significantly higher than in samples where mothers constituted more than 90% (*P* < 0.05). This difference may be attributed to the peer-support environment fostered in group-based ACT, which mitigated the lack of social support typically experienced by mothers in isolated parenting contexts [47]. Studies focusing on specific disease subgroups indicated that parents of children with autism spectrum disorder showed significant reductions in parenting stress and multidimensional improvements in psychological health following an ACT-based parenting program [42]. In contrast, parents of children with cancer experienced substantial decreases in emotional symptoms and repetitive negative thinking (RNT) through online ACT interventions [48]. These findings support the clinical adaptability of ACT in addressing diverse disease courses and stressor characteristics [49]. Moreover, the mechanism of ACT may be multimodal. At a micro level, it modulates maladaptive psychological processes such as experiential avoidance and cognitive fusion [50] while enhancing mindfulness awareness and psychological flexibility [51]. At a macro level, its structured framework integrates acceptance strategies with committed action, providing a systematic solution for managing complex stressors, such as those associated with children’s chronic illnesses [52]. Notably, the choice of intervention format requires a balance between group effects and individual differences. Although group-based ACT typically yields superior outcomes through shared experiences and synergistic strategy development [53], internet-based self-help interventions (iACT) can serve as an economically effective alternative for individuals with severe emotional disorders or limited accessibility, particularly when combined with cognitive behavioral therapy techniques to produce sustained improvements in comorbid conditions, such as post-traumatic stress disorder with chronic pain [54]. In terms of clinical practice implications, a stepped intervention strategy is recommended. Implementing high-intensity ACT courses for populations experiencing acute stress is advisable to rapidly alleviate stress symptoms. Low-density, long-term maintenance interventions are advised for individuals managing chronic stress, potentially integrating mobile health technologies such as app-based mindfulness training modules. Special attention should be given to groups with a disproportionately high proportion of mothers, for whom gender-sensitive modifications of ACT may be necessary.

## Conclusion

ACT has moderately reduced stress among parents of children with special needs (SMD =– 0.42, 95% CI– 0.72 to– 0.12, *P* = 0.0068). Subgroup analyses indicated a significant nonlinear dose-response effect of the intervention parameters: when each session lasted for at least 120 min, was administered at a frequency of at least two times per week, and extended for more than eight weeks, ACT combined with other interventions yielded the optimal stress reduction effect for parents of children with chronic or severe illnesses. Furthermore, the intervention effect in mixed-gender parent groups was significantly more significant than in samples comprising over 90% of mothers (*P* < 0.05). In terms of clinical practice implications, a stepped intervention strategy is recommended: high-intensity ACT courses should be implemented for acute stress populations to achieve rapid alleviation of stress symptoms, while for those managing chronic stress, low-density, long-term maintenance interventions—potentially integrating mobile health technologies, such as app-based mindfulness training modules—are advised. Special attention should be given to groups with a disproportionately high proportion of mothers, for whom gender-sensitive modifications of ACT may be necessary.

## Limitations

This study faced several limitations: (1) a significant degree of heterogeneity was observed among the included studies, particularly regarding intervention formats, durations, and measurement tools, which may have compromised the reliability of the pooled effect sizes; (2) there was a lack of cultural diversity, limiting the cross-cultural generalizability of the conclusions; (3) methodological flaws, such as the inadequate implementation of blinding or allocation concealment in certain studies, may have introduced bias; and (4) the majority of studies did not provide long-term follow-up data, hindering the assessment of the durability of the stress reduction effects.

## Electronic supplementary material

Below is the link to the electronic supplementary material.

Supplementary Material 1.

## Data Availability

All data and materials can be accessed by contacting the first author.
