# Supplementary material for: The effectiveness of Acceptance and Commitment Therapy on parental stress in parents of special children: a meta-analysis
Source: Child Adolesc Psychiatry Ment Health. 2025 Jul 21;19:80. doi: 10.1186/s13034-025-00944-y (PMC12278553; doi:10.1186/s13034-025-00944-y)
Supplement: Supplementary file 1 — Supplementary Material 1. [file 13034_2025_944_MOESM1_ESM.docx]

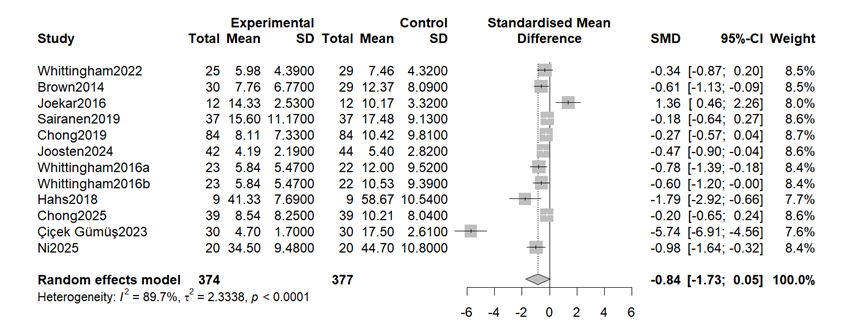


**Figure 1. Primary Forest Plot of Overall Effects**

**​**
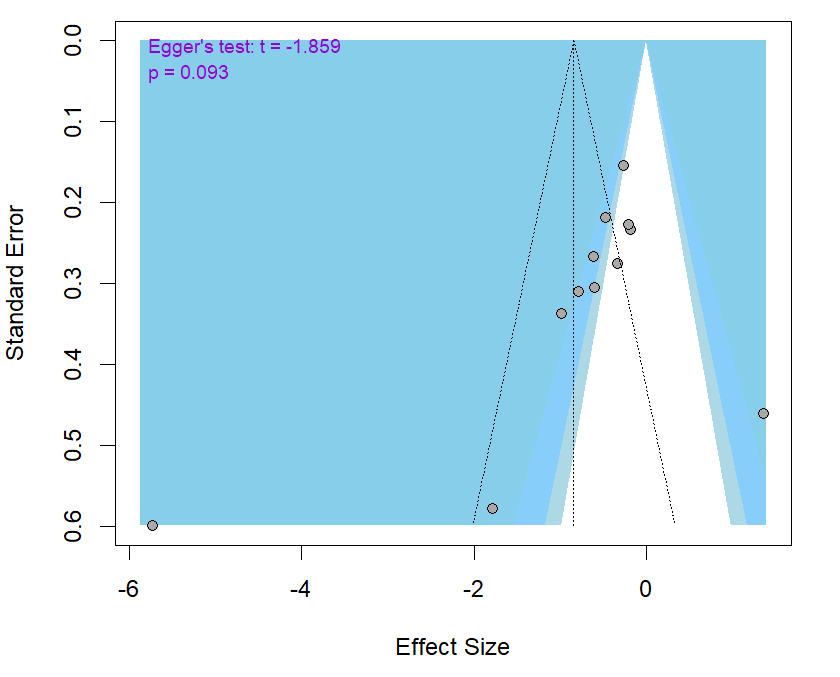


**Figure 2. Funnel Plot with Egger's Regression Test for Publication Bias**


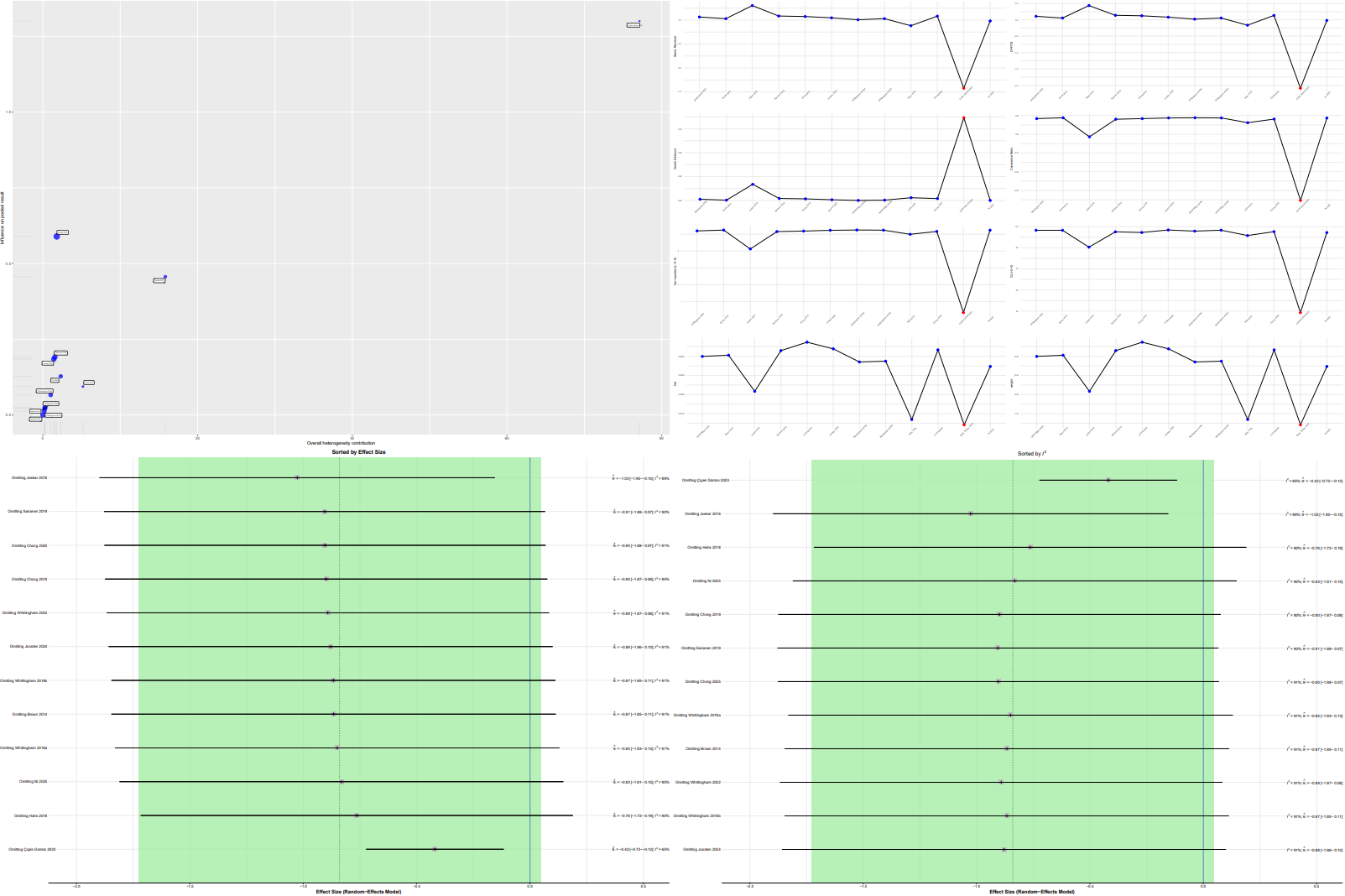


**Figure 3. Leave-One-Out Sensitivity Analysis**

**Table 2. Subgroup Analysis of Acceptance and Commitment Therapy (ACT) on Parental Stress in Parents of Children with Special Needs​**

| Dimensionality | sort | Number of studies/papers | I^2^ | Effect model | SMD and 95%CI | P |
| --- | --- | --- | --- | --- | --- | --- |
| Nation | Australia | 3 | 0.0% | Random | -0.58(-0.86,-0.30) | 0.000 |
|  | Iran | 1 | 0.0% | Random | 1.41(0.51,2.31) | 0.002 |
|  | Sweden | 1 | 0.0% | Random | -0.18(-0.64,0.27) | 0.429 |
|  | China | 3 | 55.2% | Random | -0.41(-0.79,-0.02) | 0.039 |
|  | Netherlands | 1 | 0.0% | Random | -0.48(-0.91,-0.05) | 0.029 |
|  | America | 1 | 100.0% | Random | -1.88(-3.01,-0.75) | 0.001 |
| Motion frequency | 1time/week | 6 | 75.3% | Random | -0.28(-0.73,0.18) | 0.232 |
|  | 2times/week | 3 | 66.8% | Random | -0.71(-1.22,-0.20) | 0.006 |
|  | 4times/week | 1 | 0.00% | Random | -0.21(-0.65,0.24) | 0.367 |
| Intervention cycle/week | ＜6 | 3 | 74.0% | Random | -0.53(-1.12,0.06) | 0.081 |
|  | 6-8 | 5 | 80.5% | Random | -0.36(-0.98,0.26) | 0.252 |
|  | ＞8 | 3 | 0.00% | Random | -0.36(-0.65,-0.07) | 0.014 |
| Intervention time/minute | 35 | 1 | 0.0% | Random | -0.18(-0.64,0.27) | 0.429 |
|  | 90 | 3 | 86.5% | Random | 0.07(-0.77,0.91) | 0.870 |
|  | 120 | 6 | 44.5% | Random | -0.61(-0.91,-0.32) | 0.000 |
| Subject type | Parents of children with neurodevelopmental disorders | 5 | 81.1% | Random | -0.52(-1.81,0.13) | 0.117 |
|  | Parents of chronically ill children | 3 | 0.0% | Random | -0.23(-0.45,-0.01) | 0.038 |
|  | Parents of seriously ill children | 2 | 0.0% | Random | -0.53(-0.87,-0.20) | 0.002 |
| Intervention mode | Control group no intervention | 6 | 50.0% | Random | -0.47(-0.76,-0.18) | 0.002 |
|  | Control group with intervention | 5 | 80.5% | Random | -0.27(-0.88,0.34) | 0.382 |
| Intervention mode | ACT in conjunction with other interventions | 5 | 0.0% | Random | -0.47(-0.68,-0.27) | 0.000 |
|  | Acceptance and Commitment Therapy | 5 | 84.6% | Random | -0.34(-1.01,0.33) | 0.313 |
| Parental type | Both mother and father | 6 | 54.9% | Random | -0.61(-0.92,-0.30) | 0.000 |
|  | More mothers (> 90%) | 3 | 67.3% | Random | 0.14(-0.70,0.97) | 0.746 |
| Scale type | DASS | 7 | 63.8% | Random | -0.28(-0.58,0.02) | 0.069 |
|  | Other scales | 3 | 67.3% | Random | -0.97(-1.65,-0.28) | 0.006 |

**​**

**Revised Retrieval Strategy​​**

**​​1. PubMed​​**

("Acceptance and Commitment Therapy"[Mesh] OR "ACT"[tiab] OR "acceptance and commitment therapy"[tiab])

AND

("Stress, Psychological"[Mesh] OR stress[tiab] OR "psychological stress"[tiab] OR "parental stress"[tiab] OR anxiety[tiab] OR distress[tiab])

AND

("Parents"[Mesh] OR "Caregivers"[Mesh] OR parent*[tiab] OR mother*[tiab] OR father*[tiab] OR caregiver*[tiab])

AND

("Disabled Children"[Mesh] OR "Autism Spectrum Disorder"[Mesh] OR "Cerebral Palsy"[Mesh] OR "Chronic Disease"[Mesh] OR "special needs children"[tiab] OR "children with chronic illness"[tiab] OR "children with autism"[tiab])

AND

("Randomized Controlled Trial"[pt] OR random*[tiab] OR "control group"[tiab] OR "RCT"[tiab])

NOT

("Review"[pt] OR "Case Reports"[pt] OR "Comment"[pt])

**​​2. Cochrane Library​​**

#1 [mh "Acceptance and Commitment Therapy"] OR "ACT":ti,ab,kw OR "acceptance and commitment therapy":ti,ab,kw

#2 [mh "Stress, Psychological"] OR stress:ti,ab,kw OR "psychological stress":ti,ab,kw OR "parental stress":ti,ab,kw OR anxiety:ti,ab,kw OR distress:ti,ab,kw

#3 [mh Parents] OR [mh Caregivers] OR parent*:ti,ab,kw OR mother*:ti,ab,kw OR father*:ti,ab,kw

#4 [mh "Disabled Children"] OR [mh "Autism Spectrum Disorder"] OR [mh "Cerebral Palsy"] OR [mh "Chronic Disease"] OR "special needs children":ti,ab,kw OR "children with autism":ti,ab,kw

#5 [mh "Randomized Controlled Trials"] OR "randomized controlled trial":ti,ab,kw OR RCT:ti,ab,kw

#6 #1 AND #2 AND #3 AND #4 AND #5

Limits: Publication Date to 15 April 2025, English

**​​3. Web of Science​​**

TS=("acceptance and commitment therapy" OR ACT)

AND

TS=("stress*" OR "psychological stress" OR "parental stress" OR anxiety OR distress)

AND

TS=(parent* OR mother* OR father* OR caregiver*)

AND

TS=("special needs children" OR "children with autism" OR "children with cerebral palsy" OR "children with chronic illness" OR "disabled children" OR "autism spectrum disorder" OR "chronic disease")

AND

TS=("randomized controlled trial" OR RCT OR "controlled trial")

NOT

TS=("review" OR "case report" OR "commentary")

Refined by: LANGUAGE: (English) AND DOCUMENT TYPES: (ARTICLE)

**​​4. PsycINFO​​**

1. exp "Acceptance and Commitment Therapy"/ OR ACT.mp. OR "acceptance and commitment therapy".ti,ab.

2. exp "Psychological Stress"/ OR stress.ti,ab. OR "parental stress".ti,ab. OR anxiety.ti,ab. OR distress.ti,ab.

3. exp Parents/ OR exp Caregivers/ OR parent*.ti,ab. OR mother*.ti,ab. OR father*.ti,ab.

4. exp Disabled Children/ OR exp Autism Spectrum Disorders/ OR "cerebral palsy".ti,ab. OR "chronic disease*".ti,ab. OR "special needs children".ti,ab.

5. exp Randomized Controlled Trials/ OR random*.ti,ab. OR "control group".ti,ab. OR RCT.ti,ab.

6. 1 AND 2 AND 3 AND 4 AND 5

7. limit 6 to (yr<=2025 and english language and journal article and human and adulthood (18 plus years))

8. remove duplicates from 7
